# Comparison between intervertebral oblique lumbar interbody fusion and transforaminal lumbar interbody fusion: a multicenter study

**DOI:** 10.1038/s41598-021-95774-1

**Published:** 2021-08-17

**Authors:** Hiromitsu Takaoka, Kazuhide Inage, Yawara Eguchi, Yasuhiro Shiga, Takeo Furuya, Satoshi Maki, Yasuchika Aoki, Masahiro Inoue, Takayuki Fujiyoshi, Takuya Miyamoto, Yuji Noguchi, Shinichiro Nakamura, Tomoaki Kinoshita, Takahito Kamada, Hiroshi Takahashi, Junya Saito, Masaki Norimoto, Toshiaki Kotani, Tsuyoshi Sakuma, Yasushi Iijima, Tetsuhiro Ishikawa, Tomotaka Umimura, Mitsutoshi Ohta, Miyako Suzuki-Narita, Keigo Enomoto, Takashi Sato, Masashi Sato, Masahiro Suzuki, Takashi Hozumi, Geundong Kim, Norichika Mizuki, Ryuto Tsuchiya, Takuma Otagiri, Tomohito Mukaihata, Takahisa Hishiya, Seiji Ohtori, Sumihisa Orita

**Affiliations:** 1 Department of Orthopedic Surgery, Graduate School of Medicine, Chiba University, 1-8-1 Inohana, Chuo-ku, Chiba 260-8670 Japan; 2 Center for Frontier Medical Engineering, Chiba University, Chiba, Japan; 3 Department of Orthopaedic Surgery, Eastern Chiba Medical Center, Togane, Chiba Japan; 4 Department of Orthopedic Surgery, Kimitsu Chuo Hospital, Kisarazu, Chiba Japan; 5 Department of Orthopedic Surgery, Narashino Dai-Ichi Hospital, Narashino, Chiba Japan; 6 Department of Orthopedic Surgery, University of Tsukuba, Tsukuba, Ibaraki Japan; 7 Department of Orthopaedic Surgery, Toho University Sakura Medical Center, Sakura, Chiba Japan; 8 Department of Orthopedic Surgery, Seirei Sakura Citizen Hospital, Sakura, Chiba Japan; 9 Department of Orthopedic Surgery, Sanmu Medical Center, Sanmu, Chiba Japan; 10 Department of Orthopedic Surgery, Seirei Yokohama Hospital, Yokohama, Kanagawa Japan

**Keywords:** Diseases, Neurological disorders, Spinal cord diseases

## Abstract

This study aimed to perform a comparative analysis of postoperative results between lumbar degenerative spondylolisthesis (LDS) treated with oblique lateral interbody fusion (OLIF) and transforaminal lumbar interbody fusion (TLIF) from the Chiba spine surgery registry database. Sixty-five patients who underwent single-level OLIF (O group) for LDS with ≥ 3 years’ follow-up were retrospectively reviewed. The control group comprised 78 patients who underwent single-level TLIF (T group). The analyzed variables included global alignment, radiological parameters of fused segments, asymptomatic and symptomatic ASD incidence, clinical outcomes at 3 years postoperatively using the Japanese Orthopedic Association Back Pain Evaluation Questionnaire data, visual analogue scale scores for low back pain, lower extremity pain, and lower extremity numbness. There was no significant change in global alignment between the two groups. The rate of improvement in anterior intervertebral disc height was not significantly different between the groups at 1-month postoperatively. However, at the final evaluation, the anterior intervertebral disc height and incidence of asymptomatic ASD were significantly higher in the O group. There was no significant difference in symptomatic ASD, reoperation cases, or clinical results between groups. Thus, single-level OLIF can maintain the corrected disc height, but as it has no effect on global alignment, its benefit is limited.

The posterior approach of posterior lumbar interbody fusion (PLIF) and transforaminal lumbar interbody fusion (TLIF) has been used for the treatment of spinal canal stenosis associated with lumbar degenerative spondylolisthesis (LDS)^1–3^. Oblique lateral interbody fusion (OLIF), in contrast, has recently been adopted by spine surgeons as a minimally invasive and rigid fixation procedure using a lateral approach to insert a large cage between the vertebrae to improve spinal alignment. OLIF has been used in the treatment of LDS and its indirect decompression effect results in progressive widening of the dural canal, resulting in improvement of neurological symptoms in the lower extremities^4,5^. However, this technique involves the insertion of a cage with a height larger than the preoperative disc height to achieve indirect decompression, which may have an impact on the adjacent vertebrae. Few reports on the incidence of adjacent segment disorder (ASD) in OLIF have been published or compared with other interbody fusion procedures. The purpose of this study was to extract cases from the Chiba spine surgery registry (CSSR) database in which single-level OLIF and conventional single-level TLIF were performed for LDS and perform a comparative analysis of changes in global alignment, incidence of ASD, and clinical outcome 3 years after surgery.

## Patients and methods

This was a multicenter, retrospective, observational study conducted with the approval of the Ethics Committee at our institution. The study protocol was approved by the ethics review committee of School of Medicine, Chiba University (IRB approval code: 2030). The opt-out method was adopted to obtain informed consent from the patients. Among 14,267 patients enrolled in the CSSR database since 2012, patients with lumbar spinal canal stenosis associated with LDS and neurological symptoms in the lower extremities who received single-level OLIF (group O) and single-level TLIF (group T) were included in this study. Exclusion criteria were: patients who underwent fusion of ≥ 2 vertebrae and combined surgery with other fusions. Patients followed up for 3 years after surgery were selected for this retrospective study. The O group consisted of 65 patients (27 men and 38 women, mean age 66 ± 12 years), and the T group of 78 patients (36 men and 42 women, mean age 71 ± 9 years), with a mean follow-up period of 53 ± 13.0 months (Table 1).

**Table 1:** Demographic characteristics.

	O group	T group	*P* value
Age, y.o	66 ± 12	71 ± 9	0.122
Sex	Male 28 Female 38	Male 37 Female 42	0.595
Fusion level	L3/4 13	L3/4 8	0.109
(Cases)	L4/5 53	L4/5 70	
Follow up (months)	64.0 ± 16.2	53.0 ± 13.0	< 0.001
**Preoperative parameter**			
LL, °	36 ± 14	40 ± 14	0.127
PT, °	22 ± 10	22 ± 7	0.42
PI-LL, °	15 ± 14	13 ± 13	0.347
SS, °	29 ± 11	30 ± 8	0.501
**Local parameter**			
Anterior disc height, mm	8.2 ± 3	7.9 ± 2.5	0.483
Posterior disc height, mm	5.1 ± 2.1	4.6 ± 1.9	0.097
Segmental lordotic angle, °	15.6 ± 6.5	13.7 ± 6.5	0.123

LL indicates lumbar lordosis; *PT* pelvic tilt, *PI-LL* pelvic incidence minus lumbar lordosis, *SS* sacral slope.

### Evaluation items

#### Image assessment

##### Global alignment

All patients underwent upright lumbar digital radiography in the frontal, lateral, and anterior flexion positions preoperatively, 1 month postoperatively, and 3 years postoperatively. Lumbar lordosis (LL), pelvic tilt (PT), sacral slope (SS), and pelvic incidence (PI) were measured on lateral digital radiographic images. Changes in LL, PT, and PI-LL were investigated preoperatively to 1 month and 3 years postoperatively.

##### Radiological parameters of fused segments

The anterior and posterior intervertebral heights and local angles of the fixed intervertebral space were measured and compared with those of the O and T groups. The local angle was defined as the angle between the caudal endplate of the cephalad vertebral body and the cephalad endplate of the caudal vertebral body.

##### Asymptomatic ASD incidence

Asymptomatic ASD was defined as (1) a decrease in disc height > 3 mm, (2) slippage progression > 5%, and (3) a posterior widening > 5° according to Okuda et al.^6^. The incidence of ASD in the upper fixed vertebrae 3 years after surgery was investigated. The change from the preoperative period to 3 years after surgery was assessed in each group, and the rate of change between the two groups compared.

##### Symptomatic ASD incidence

Symptomatic ASD was defined as the presence of clinical symptoms of ASD at the time of the final assessment. The incidence of symptomatic ASD and reoperation rate during follow-up were investigated and compared between both groups.

#### Clinical results

##### Visual analog scale (VAS)

Low back pain VAS (LBP-VAS), lower extremity pain VAS (BLP-VAS), and lower extremity numbness VAS (BLN-VAS) were evaluated preoperatively and at 3 years postoperatively as postoperative clinical outcome assessment.

#### JOABPEQ

The efficacy rates of each domain in the Japanese Orthopedic Association Back Pain Evaluation Questionnaire (JOABPEQ)^7^ were used to compare the preoperative and 3-year postoperative surveys.

### Statistical analysis

For statistical analysis, the Mann–Whitney U test was used for each preoperative assessment in the O and T groups; the Student's t-test for pre- and postoperative imaging and spinal-pelvic parameters, and the Chi-squared test for the ASD and the JOABPEQ efficacy rate comparison between the O and T groups in each domain. In each domain, if more than one of the following conditions was met, it was judged as "effective”: when the post-operative score obtained increased by ≥ 20 points compared to the pre-operative score, when the value of the preoperative score was < 90 points, and when the post-treatment reached a value ≥ 90 points.

The percentage of patients where the treatment was judged to be "effective" was then defined as the effective rate, and calculated for each domain.

#### Surgical technique: single-level OLIF

**Figure 1:**
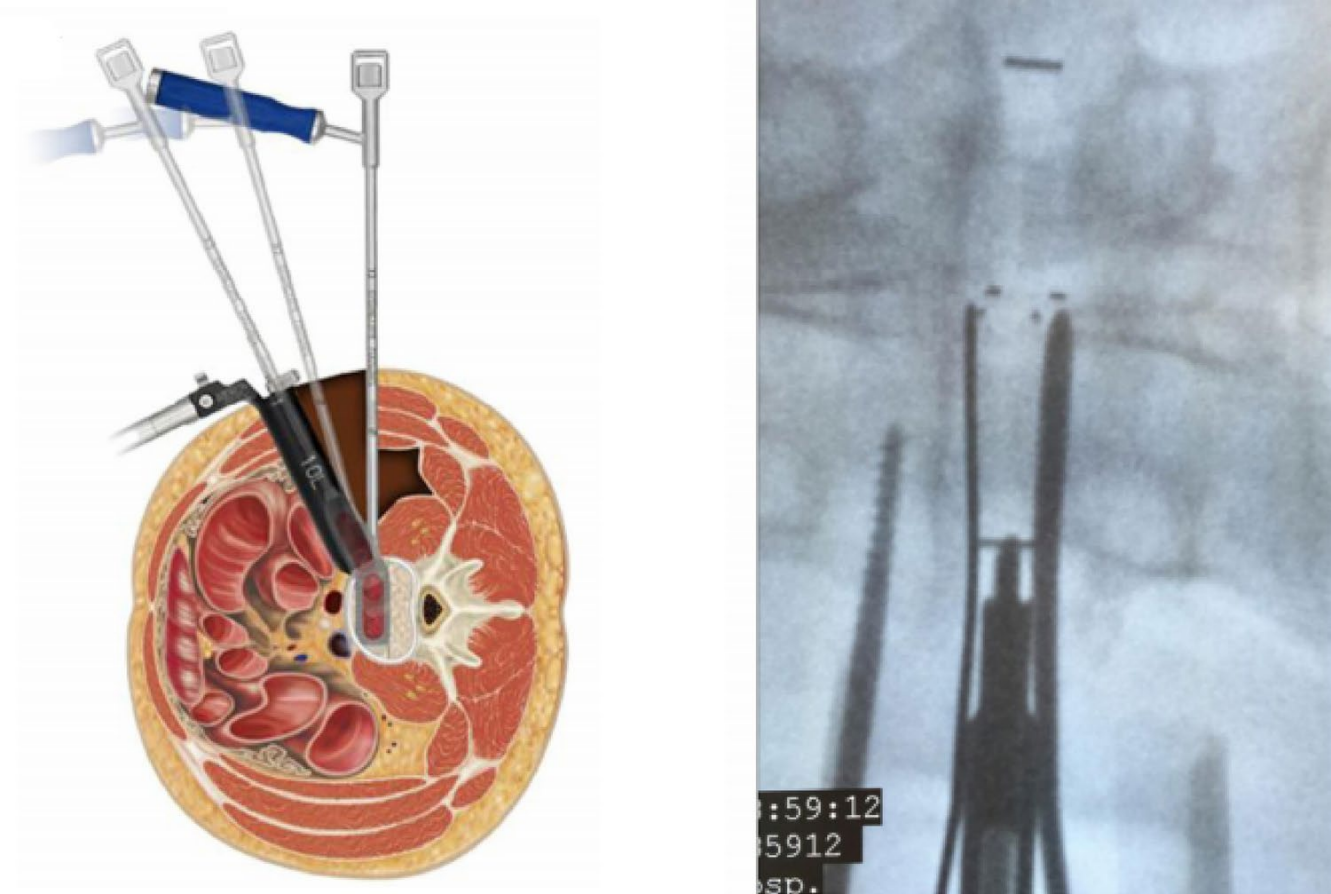
When inserting the cage, the inserter enters it obliquely and then turns it orthogonally to allow the surgeon to place the cage orthogonally across the disc space.

All patients initially underwent OLIF surgery. The patients were placed in the lateral decubitus position with their left side up. The target intervertebral disc space was identified, the midportion of the disc space marked, and a 4-cm skin incision performed. After dissection and approach to the retroperitoneal space, a finger was used to sweep the peritoneal contents and the retroperitoneal fat anteriorly past the anterior portion of the psoas. Then, the intervertebral disc was expanded from the front of the psoas and the retractor opened to expose the disc. The cage size was determined by trial, and an appropriately sized static cage placed (Fig. 1). After cage placement, each patient was repositioned prone onto a frame. Then, small paramedian incisions were made, and percutaneous pedicle screw-and-rod placement was performed. No patient underwent posterior decompression.

#### Surgical technique: single-level TLIF

Although the surgery was performed through a midline incision, the approach was based on the surgeon’s preference. A laminectomy and unilateral facetectomy were performed, followed by discectomy, and endplates were curetted and prepared. Disc space distraction was also performed with intradiscal spreaders before the placement of local autogenous bone graft and an interbody cage. The cage type was chosen according to the surgeon's preference. Thereafter, bilateral pedicle screw-rod constructs were inserted.

### Ethics and consent to participate

This multicenter, retrospective, observational study declares that all protocols were conducted in accordance with the ethical standards set forth in the 1964 Declaration of Helsinki and subsequent amendments, and were approved by the Ethics Committee of this hospital. We declare that all participants provided written informed consent before their inclusion in this study.

## Results

### Image evaluation

#### Global alignment

There was no significant change in global alignment between the O and T groups 1 month after surgery or at the final evaluation (Table 2).

**Table 2:** Average change of spinal parameter.

	Pre-op	Post-op 1 m	Post op 3 yrs	*P* value
**O group**				
LL, °	36 ± 14	37 ± 14	37 ± 17	NS
PT, °	22 ± 10	20 ± 10	21 ± 9	NS
SS, °	29 ± 11	31 ± 10	30 ± 10	NS
PI-LL, °	15 ± 14	14 ± 13	13 ± 15	NS
**T group**				
LL, °	40 ± 14	41 ± 13	41 ± 15	NS
PT, °	22 ± 7	23 ± 8	22 ± 8	NS
SS, °	30 ± 8	30 ± 8	30 ± 9	NS
PI-LL, °	13 ± 13	12 ± 13	11 ± 14	NS

Pre-op indicates preoperative; Post-op1m, postoperative 1 month; Post-op 3yrs, postoperative 3 years.

#### Radiological parameters of fused segments

**Figure 2:**
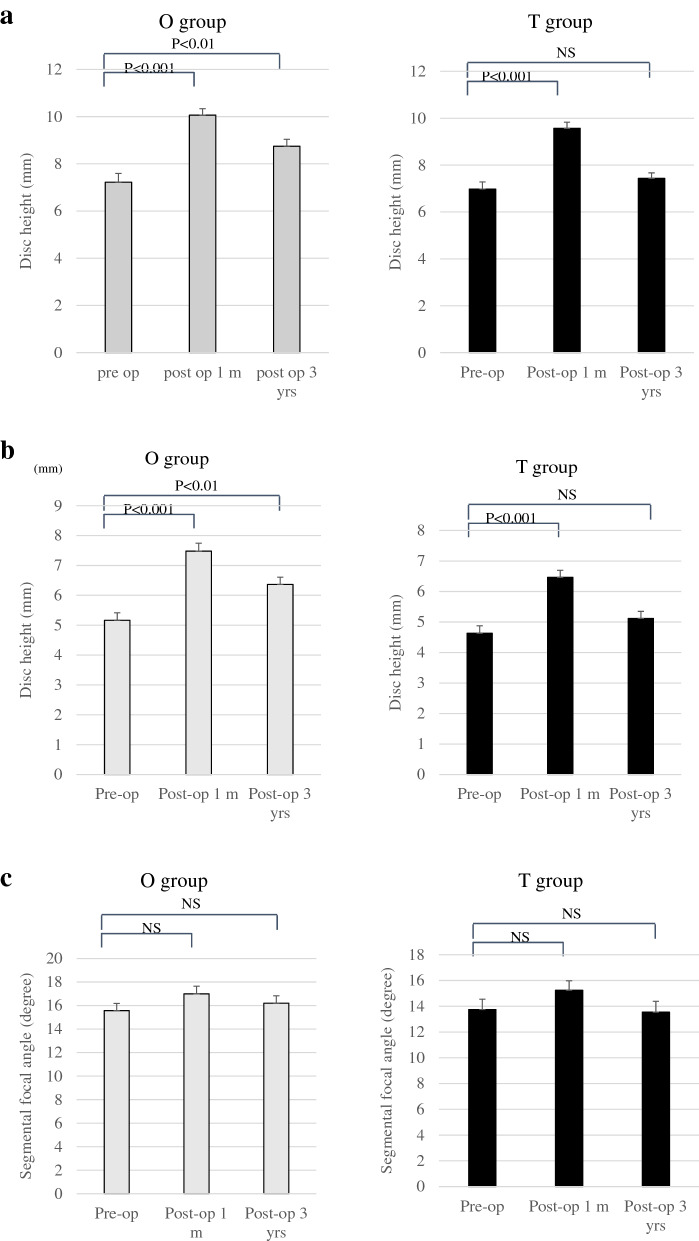
(**a**) Average change of anterior disc height. The anterior intervertebral height in the O group was significantly higher than that preoperatively. The anterior intervertebral height of the T group was significantly higher at one month compared with the preoperative level, but not at 3 years. (**b**) Average change of posterior disc height. The posterior intervertebral height shows the same trend as the anterior one. (**c**) Improvement rate of segmental focal angle. There was no significant difference in focal angle between the two groups at any time point. Abbreviations: T group, patients who underwent single-level transforaminal lumbar interbody fusion; O group, patients who underwent single-level oblique lateral interbody fusion.

**Figure 3:**
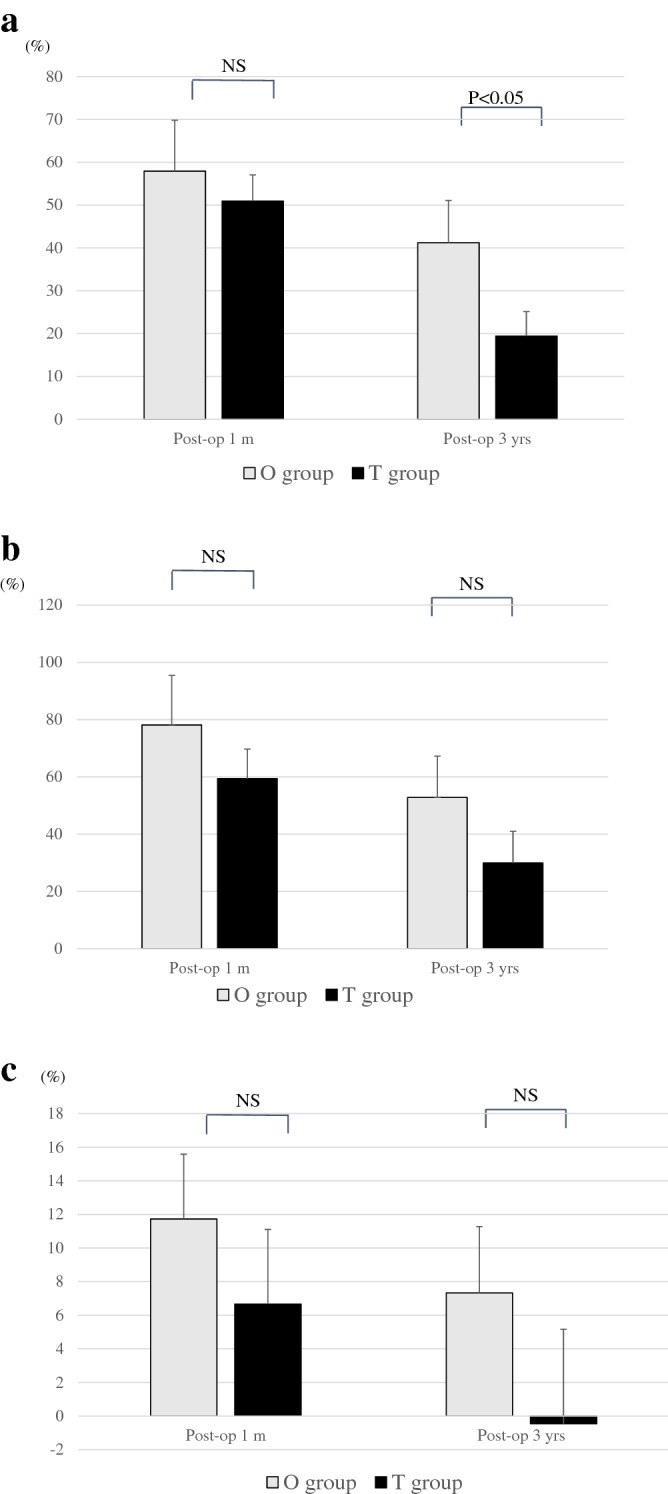
(**a**) Improvement rate of anterior disc height. The rate of improvement in the anterior intervertebral disc height was significantly higher in the O group. (**b**) Improvement rate of posterior disc height. (**c**) Improvement rate of segmental focal angle. There was no significant difference in the posterior disc height and rate of improvement between the O and T groups at one month postoperatively and at the final evaluation. Abbreviations: T group, patients who underwent single-level transforaminal lumbar interbody fusion; O group, patients who underwent single-level oblique lateral interbody fusion.

The anterior intervertebral height in the O group was significantly higher than that measured preoperatively (*P* < 0.001 for preoperative vs. 1 month, *P* < 0.05 for preoperative vs. 3 years) (8.2 ± 3.0 mm preoperatively, 11.0 ± 2.2 mm at 1 month, and 9.7 ± 2.4 mm at 3 years postoperatively). The anterior intervertebral height of the T group was significantly higher (*P* < 0.001) at 1 month than that at the preoperative level, but not at 3 years (7.0 ± 2.5 mm preoperatively, 9.6 ± 2.2 mm at 1 month, and 7.4 ± 2.0 mm at 3 years after the surgery). The posterior intervertebral height showed the same trend as the anterior one. The postoperative posterior intervertebral height in the O group was significantly higher than that preoperatively (preoperative vs. 1 month postoperatively: *P* < 0.001, preoperative vs. 3 years postoperatively: *P* < 0.05) (5.1 ± 2.0 mm preoperatively, 7.5 ± 2.1 mm 1 month postoperatively, 6.3 ± 1.9 mm 3 years postoperatively). In contrast, the postoperative posterior intervertebral height of the T group was significantly higher (*P* < 0.001) at 1 month, but not at 3 years (4.6 ± 2.0 mm preoperatively, 6.5 ± 1.9 mm at 1 month, and 5.1 ± 1.9 mm at 3 years) than that measured preoperatively. There was no significant difference in the focal angle between the two groups at any time point (Fig. 2). Second, the improvement rate in anterior intervertebral disc height was not significantly different between the two groups 1 month postoperatively; however, at the final evaluation, the anterior intervertebral disc height was significantly higher in the O group than in the T group (*P* < 0.05). There was no significant difference in posterior disc height or rate of improvement between groups at 1 month after surgery and at the final evaluation (Fig. 3).

#### Asymptomatic ASD incidence

**Figure 4:**
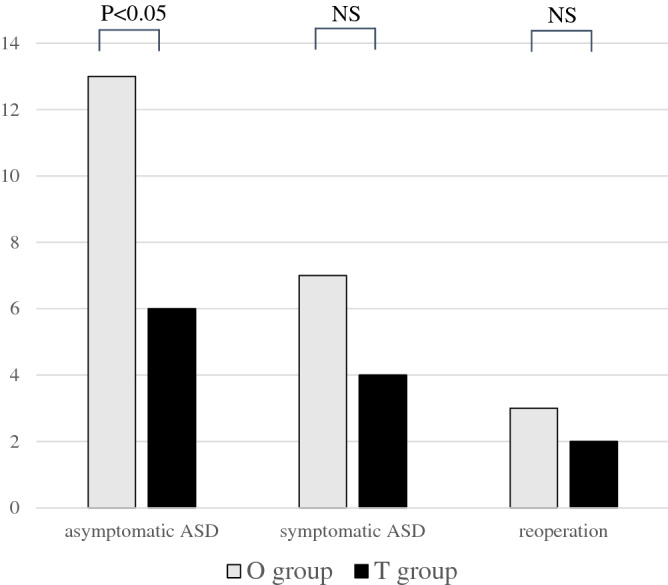
Frequency of complications. The frequency of asymptomatic ASD was significantly higher in the O group. There was no significant difference in symptomatic ASD between the O group and T group, and no significant difference in reoperation cases between the O group and T group. Abbreviations: T group, patients who underwent single-level transforaminal lumbar interbody fusion; O group, patients who underwent single-level oblique lateral interbody fusion; ASD, adjacent segment disorder.

The frequency of asymptomatic ASD was 21.6% in the O group and 6.8% in the T group, with a significantly higher incidence in the O group than in the T group (*P* < 0.05) (Fig. 4).

#### Symptomatic ASD incidence

There was no significant difference in symptomatic ASD between the O group (7 cases, 10.8%) and T group (4 cases, 5.1%), or in reoperation cases (O: 3 cases, 4.6%, P = 0.21 and T: 2 cases, 2.6%, *P* = 0.51; Fig. 4).

### Clinical results

#### VAS

The LBP-VAS decreased significantly from 50.8 to 19.0 in the O group (*P* < 0.001) and from 58.2 to 18.8 in the T group (*P* < 0.001). The BLN-VAS value significantly decreased from 62.0 to 22.8 in the O group (*P* < 0.001), and from 79.8 to 18.8 in the T group (*P* < 0.001) (Table 3).

**Table 3:** Change of visual analog scale.

	Pre-op	Post-op 3 yrs	*P* value
**O group**			
LBP-VAS	50.8 ± 6.5	19 ± 4.4	< 0.001
BLP-VAS	73.6 ± 4.7	23.5 ± 5.2	< 0.001
BLN-VAS	67.7 ± 5.1	20.9 ± 3.9	< 0.001
**T group**			
LBP-VAS	58.2 ± 6.3	18.8 ± 4.8	< 0.001
BLP-VAS	76.3 ± 3.9	12.8 ± 3.9	< 0.001
BLN-VAS	78.8 ± 3.2	19.7 ± 5.4	< 0.001

Pre-op indicates preoperative; Post-op 3yrs, postoperative 3 years.

#### JOABPEQ

There was no significant difference in the preoperative JOABPEQ scores between the two groups (Table 4). The therapeutic effectiveness of the JOABPEQ domains in the O group 3 years after surgery was 80.9% for low back pain, 71.0% for lumbar function, 80.1% for walking ability, 62.3% for social life function, and 32.8% for mental health. On the other hand, in the T group, low back pain was 88.1%, lumbar function 65.9%, walking ability 85.3%, social life function 68.2%, and mental health 41.8% in each domain. There was no significant difference in therapeutic effectiveness (Table 5).

**Table 4:** Preoperative the Japanese orthopedic association back pain evaluation questionnaire scores.

	O group	T group	*P* value
Low back pain	51 ± 7	39 ± 4	0.18
Lumbar function	58 ± 6	54 ± 4	0.64
Walking ability	27 ± 4	25 ± 3	0.71
Social life function	42 ± 5	43 ± 3	0.87
Mental health	50 ± 3	43 ± 3	0.18

**Table 5:** Therapeutic effectiveness JOABPEQ.

	O group	T group	*P* value
Low back pain, %	81	88	0.28
Lumbar function, %	70	66	0.65
Walking ability, %	80	85	0.42
Social life function, %	62	68	0.54
Mental health, %	33	42	0.33

There was no significant difference in therapeutic effectiveness between the two groups.

Total complications were 22 in the O group and 13 in the T group (*P* = 0.77). The most common complication in the O group was endplate fracture/subsidence in 11 cases, followed by transient weakness of the psoas muscle and thigh numbness in 10 cases, and segmental artery injury in 1 case. Almost all of these complications were transient; in the T group, there were 6 cases of lower extremity numbness, 3 of asymptomatic screw malposition, 2 of dural tear, 1 of postoperative hematoma, and 1 of postoperative infection.

## Discussion

In recent years, the outcomes of various techniques for intervertebral fusion surgery have been compared^8–12^. The main purpose of this study was to compare the global alignment, intervertebral height, and incidence of ASD between OLIF and TLIF procedures.

### Image evaluation

#### Global alignment

In the present study, there was no significant improvement in the global alignment of single-level OLIF and single-level TLIF. In a previous study, Champagne et al.^11^ compared OLIF and TLIF techniques before and after surgery for global alignment and reported a better degree of improvement in alignment in OLIF, but mainly in cases with multiple intervertebral joints. On the other hand, Saadeh et al.^8^ and Jin et al.^13^ found no significant difference in the improvement of alignment in TLIF and OLIF when the fixed intervertebral space was limited to a single-level space, as single-level fixation had less impact on overall alignment. In the present study, intervertebral fusion was limited to a single-level space, and our results were similar to those of Saadeh et al. Another reason may be that the treatment concept differed from the purpose of alignment improvement in this study.

#### Radiological parameters of fused segments

Postoperative intervertebral height was found to remain significantly higher in OLIF until the final evaluation. Endplate subsidence has been reported to be 30–70% for TLIF^14–18^ and 11–30% for OLIF^19–22^, According to a biomechanical study by Lu et al.^10^, as the surface area of the OLIF cage is much larger than that of the TLIF cage, the stresses on the vertebral endplates and cancellous bone are distributed and less susceptible to subsidence. Lin et al.^4^ also reported a higher cage subsidence rate in minimally invasive (MI) TLIF than in OLIF because the OLIF cage is placed at the strongest point on both ends of the vertebral endplate, whereas the TLIF cage is mostly placed near the center of the vertebral endplate. The results of the present study were similar to those of Lin et al., with OLIF having a lower risk of cage subsidence than TLIF and an advantage in maintaining local alignment.

#### Incidence of asymptomatic ASD

Risk factors for the occurrence of ASD were length of postoperative spine follow-up, alignment and instrumentation length, and fixation focal stiffness^23–29^. In this study, there was no difference in the length of postoperative follow-up, alignment, and instrumentation between groups. Regarding the stiffness of fixed locations, Lin et al.^4^ reported that OLIF allowed insertion of a larger cage than TLIF, as previously mentioned. In other words, the load on the adjacent vertebrae may be increased by OLIF because of its higher stiffness. Therefore, the results of this study suggest that OLIF may increase the incidence of asymptomatic ASDs due to its superior local stiffness.

#### Symptomatic ASD incidence

To the best of our knowledge, there are no reports describing the incidence of symptomatic ASD in OLIF and TLIF, and those describing cases of symptomatic ASD in anterior lumbar interbody fusion (ALIF) and TLIF present medium to long-term results. The incidence of symptomatic ASD after a mean follow-up > 3 years has been reported to range from approximately 0 to 14.7% for ALIF and from about 1.3% to 19% for TLIF^24,25,30,31^. Bae et al.^30^ investigated 103 patients with ALIF and TLIF, followed up for > 3 years, and compared their clinical results. At a mean 59.1 months of follow-up, there was no significant difference between the two techniques. The results of the present study suggest that the progression to symptomatic ASDs may follow a similar process for OLIF and TLIF.

### Clinical results

#### VAS, JOABPEQ

In the present study, there was no significant difference between the two groups in terms of changes in the VAS values and the clinical outcome assessment of each JOABPEQ domain > 3 years after surgery. OLIF is a mechanism of indirect decompression, which differs from TLIF in terms of direct decompression. Sato et al.^5^ reported that OLIF significantly improved postoperative clinical outcomes due to indirect decompression, which increases the interdural area as a result of higher disc height. Koike et al.^32^ reported a significant improvement in postoperative clinical outcomes in the first year after surgery. They reported that the effect of indirect decompression of OLIF was comparable to that of direct TLIF decompression. The present study showed that OLIF was as effective as TLIF in improving clinical outcomes for > 3 years after surgery.

## Limitations

This study has several limitations. First, we did not evaluate the presence or absence of decompression between adjacent vertebrae in a unified manner. Second, there were some differences in the patient backgrounds and results between the two groups owing to the use of registry data. Third, this was a small study, and some parameters that were not significantly different with the current sample size may become significant with higher number of cases. Fourth, the same cage was used for all cases in OLIF, but the cage used in TLIF differed from one facility to another.

## Conclusion

In the present study, single-level OLIF for LDS resulted in a significantly higher fixed disc height compared to single-level TLIF, but a higher incidence of asymptomatic ASD. There was no difference between the two groups in terms of pre- and postoperative spinal and pelvic parameters in single-level fixation. Clinical outcomes did not differ between OLIF and TLIF, and the effect of indirect decompression of OLIF and that of posterior direct decompression of TLIF were comparable. In single-level interbody fusion, OLIF was found to be more suitable for maintaining the corrected disc height than TLIF; however, since it had no effect on global alignment, its benefit is limited. Additionally, there is a concern about the incidence of asymptomatic ASD.

## Data Availability

The datasets generated during and/or analyzed during the current study are available in the Pub Med.
